# Deep Untargeted Metabolomics Analysis to Further Characterize the Adaptation Response of *Gliricidia sepium* (Jacq.) Walp. to Very High Salinity Stress

**DOI:** 10.3389/fpls.2022.869105

**Published:** 2022-05-19

**Authors:** Ítalo de Oliveira Braga, Thalliton Luiz Carvalho da Silva, Vivianny Nayse Belo Silva, Jorge Candido Rodrigues Neto, José Antônio de Aquino Ribeiro, Patrícia Verardi Abdelnur, Carlos Antônio Ferreira de Sousa, Manoel Teixeira Souza

**Affiliations:** ^1^Graduate Program of Plant Biotechnology, Federal University of Lavras, Lavras, Brazil; ^2^Institute of Chemistry, Federal University of Goiás, Campus Samambaia, Goiânia, Brazil; ^3^Brazilian Agricultural Research Corporation, Embrapa Agroenergy, Brasília, Brazil; ^4^Brazilian Agricultural Research Corporation, Embrapa Mid-North, Teresina, Brazil

**Keywords:** abiotic stress, salt tolerance, chemometrics, high-resolution mass spectrometry, phenylpropanoids, phytosterols, lignin

## Abstract

The multipurpose tree *Gliricidia sepium* (Jacq.) Walp. adapts to a very high level of salt stress (≥20 dS m^−1^) and resumes the production of new leaves around 2 weeks after losing all leaves due to abrupt salinity stress. The integration of metabolome and transcriptome profiles from gliricidia leaves points to a central role of the phenylpropanoid biosynthesis pathway in the short-term response to salinity stress. In this study, a deeper untargeted metabolomics analysis of the leaves and roots of young gliricidia plants was conducted to characterize the mechanism(s) behind this adaptation response. The polar and lipidic fractions from leaf and root samples were extracted and analyzed on a UHPLC.ESI.Q-TOF.HRMS system. Acquired data were analyzed using the XCMS Online, and MetaboAnalyst platforms, *via* three distinct and complementary strategies. Together, the results obtained first led us to postulate that these plants are salt-excluding plants, which adapted to high salinity stress *via* two salt-excluding mechanisms, starting in the canopy—severe defoliation—and concluding in the roots—limited entry of Na. Besides that, it was possible to show that the phenylpropanoid biosynthesis pathway plays a role throughout the entire adaptation response, starting in the short term and continuing in the long one. The roots metabolome analysis revealed 11 distinct metabolic pathways affected by salt stress, and the initial analysis of the two most affected ones—steroid biosynthesis and lysine biosynthesis—led us also to postulate that the accumulation of lignin and some phytosterols, as well as lysine biosynthesis—but not degradation, play a role in promoting the adaptation response. However, additional studies are necessary to investigate these hypotheses.

## Introduction

Soil salinity is an environmental limiting factor for plant biomass production worldwide, with approximately 20% of all agricultural land in the world having either saline or sodic soils, and between 25 and 30% of the irrigated land area is affected by salt (Negrão et al., 2017; Shahid et al., 2018). The annual global cost of salt-induced land degradation in irrigated areas can reach US$ 27.3 billion due to a decrease in productivity (Pan et al., 2020). Salinity imposes adverse effects on plant growth by causing water imbalance, oxidative stress, and Na^+^ toxicity (Zarei et al., 2020). Besides that, it also compromises germinative processes, photosynthetic pigmentation, and photosynthesis.

Metabolomics (the study of modification in metabolites) is a comprehensive and quantitative analysis of all small molecules in a biological system (Belinato et al., 2019). It is one promising approach used to detect and quantify primary and secondary metabolites of low molecular weight, generally <1,500 Da (Bueno and Lopes, 2020). Recent studies show that some metabolites are present in metabolic changes induced by salt stress, and they can act as effectors of osmotic readjustment or antioxidant response (Arbona et al., 2013). The presence of specific metabolites may be associated with tolerance to stress and serve as biomarkers for salt-tolerant genotypes selection in plant breeding programs (D'amelia et al., 2018).

*Gliricidia sepium* (Jacq.) Walp., a medium-sized legume (10–15 m) from the Fabaceae family, is originally from Central America. It is one of the most well-known multipurpose trees, known for its ability to adapt very well to a wide range of soils, from eroded acidic soils, sandy soils, heavy clay, limestone, and alkaline soils (Rahman et al., 2019). As pointed out by Rahman et al. (2019), Gliricidia salinity tolerance limits alongside the morphophysiological responses to salt stress are not yet well-understood. Rahman and colleagues reported a study where seawater from the southern coastal area of Bangladesh induced salinity stress in 1-month-old gliricidia seedlings for 90 days and showed that seawater-induced salinity negatively affected several growth-related attributes. They also showed enhanced accumulation of proline, the proteinogenic secondary amino acid that participates in metabolic signaling and is known to be metabolized by its own family of enzymes responding to stress (Phang et al., 2010), postulating that it might help adjust the plant to water deficit conditions (Rahman et al., 2019).

In a previous study done by our group, we described two distinct responses of gliricidia plants—tolerance and adaptation—to salt stress, depending on the amount of NaCl used (Carvalho da Silva et al., 2021). Additionally, when employing single and integrative transcriptomic and metabolomic analysis approaches, it showed that the phenylpropanoid biosynthesis pathway was the most salt stress-affected pathway in the leaves of young gliricidia plants, with 15 metabolites and three genes differentially expressed, and that this pathway role was more evident at the beginning of the stress, not in the long-term.

Ho et al. (2020) characterized the transcriptomes, metabolomes, and lipidomes of domesticated and landrace barley plants with distinct seedling root growth responses under salt stress. The phenylpropanoid biosynthesis was the most statistically enriched biological pathway among all salinity responses observed, based on pathway over-representation of the differentially expressed genes and metabolites (Ho et al., 2020). Zhu et al. (2021) also performed a combined transcriptomic and metabolomic analysis of salt-stressed *Sophora alepecuroide* plants, a leguminous perennial herb found mainly in the desert and semi-desert areas of the China northwest region, and also showed that the differentially expressed genes and metabolites in the phenylpropanoid biosynthesis pathway significantly correlated under salt stress. A difference between the work done by Carvalho da Silva et al. (2021) and the ones by Ho et al. (2020) and Zhu et al. (2021) is that it used leaves and the later ones used roots.

The current study is a follow-up to our previous study (Carvalho da Silva et al., 2021). Hence, the objective of this present study was to carry out a deeper metabolome analysis not only of the leaves but also the roots of *G. sepium* plants submitted to very high salt stress. For this purpose, gliricidia plants were under salinity stress, and leaves and root samples were collected from control and stressed plants at 2 and 55 days after the onset of the stress for untargeted metabolomics (UM) analysis.

## Materials and Methods

### Plant Material, Growth Conditions, Experimental Design, and Saline Stress

The accession of gliricidia [*Gliricidia sepium* (Jacq.) Steud.] used in this study belongs to the Gliricidia Collection at Embrapa Tabuleiros Costeiros (www.embrapa.br/en/tabuleiros-costeiros). After soaking the seeds in 2% sodium hypochlorite and Tween® 20 for 5 min under slow agitation, we washed them with sterile water and dried them on sterilized filter paper. Then they were placed in a Petri dish with filter paper moistened with sterilized water until the radicle emission. Subsequently, each germinated seed was transferred individually to a 5 L plastic pot (about 20 cm × 25 cm × 15 cm in size, top × height × bottom) containing 4 kg of substrate previously prepared by mixing sterile soil, vermiculite, and a commercial substrate (Bioplant®), in the ratio 2/1/1 (v/v/v); and kept in a greenhouse for 3 months.

Groups of five and a half-month-old gliricidia plants were kept under control conditions or subjected to saline stress (27 dS/m of electric conductivity) for 2 (short-term stress) or 55 (long-term stress) days. The experimental design was completely randomized with 12 replicates (plants) per treatment.

The NaCl was dissolved in deionized water to salinize the substrate. We replaced the water lost by evapotranspiration with deionized water on a daily basis, and the amount of deionized water used corresponded to the difference between the amount of water previously present in the substrate and the amount of water necessary for the substrate to reach field capacity, as described in Vieira et al. (2020); Carvalho da Silva et al. (2021). Applying the right amount of water—up to the substrate field capacity—was a means of ensuring no leakage of the solution out of the pot and no loss of Na^+^ or Cl^−^. The electric conductivity and water potential in the substrate solution were monitored at 0 and 25 days after imposing the stress treatment for all replicates. The electric conductivity in the soil was measured at field capacity (ECfc) and not in the saturated paste extract (ECe), as also described in (Vieira et al., 2020; Carvalho da Silva et al., 2021).

### Mineral Analysis

Samples were collected for determination of mineral content; as well as samples of the substrate before and after plant growth. Dried samples were ground in a Wiley mill Tecnal Mod. TE 680 (Tecnal, Piracicaba, SP, Brazil), passed through a 1 mm (20 mesh) sieve and then subjected to the extraction of minerals by the standard methods used in laboratory routine at Soloquímica (www.soloquimica.com.br).

Initially, mineral analysis data were examined for normality using the Shapiro-Wilk test. Then, parametric and non-parametric data were compared using the *T*-tests (*p* < 0.05) and Mann-Whitney test (*p* < 0.05), respectively, and biomass data were examined by linear regression analysis.

### Metabolomics Analysis

Leaves and roots from control and stressed plants—five replicates per treatment—were collected at 2 and 55 days after stress treatment (DAT), immediately immersed in liquid nitrogen, and then stored at −80°C until extraction of metabolites.

#### Chemicals and Metabolites Extraction

Samples were grounded in liquid nitrogen before solvent extraction. The solvents methanol grade ultra-high performance liquid chromatography (UHPLC), acetonitrile grade liquid chromatography-mass-spectrometry (LC-MS), formic acid grade LC-MS and sodium hydroxide American Chemical Society (ACS) grade LC-MS were from Sigma-Aldrich (St. Louis, MO, USA), and the water was treated in a Milli-Q system (Millipore, Bedford, MA, USA).

We employed a well-known protocol (Vargas et al., 2016; Rodrigues-Neto et al., 2018) to extract the metabolites through a mixture of polar solvents. After transferring aliquots of 50 mg of grounded sample to 2 ml microtubes, 1 ml of a 1:3 (v:v) methanol: methyl tert-butyl ether mixture was added, and then left for homogenization at 4°C on an orbital shaker for 10 min, followed by an ultrasound treatment in an ice bath for another 10 min. Next, 500 μl of a 1:3 (v:v) methanol: water mixture (1:3) was added to each microtube before centrifugation (12,000 rpm, 4°C for 5 min). After centrifugation, three phases were obtained: a non-polar phase for lipid analysis, a polar phase for secondary metabolism analysis and a pellet for protein analysis. The lipidic and polar fractions were transferred separately to 1.5 ml microtubes and vacuum dried overnight in room temperature in a Speed vac system (Centrivap, Labconco, Kansa, MO, USA). Polar and lipidic fractions samples were resuspended in a 500 μl of 1:3 (v:v) methanol: water mixture and transferred to vials prior to chemical analysis.

#### UHPLC-MS and UHPLC-MS/MS

A UHPLC chromatographic system was used (Nexera X2, Shimadzu Corporation, Japan) equipped with an Acquity UPLC HSS T3 (1.8 μm, 2.1 × 150 mm) reverse phase column (Waters Technologies, Milford, MA), maintained at 35°C. A polar mobile phase was used, composed by water (solvent A) and acetonitrile/methanol (70/30, v/v) (solvent B), both in 0.1% formic acid. The gradient elution used, with a flow rate of 0.4 mL min^−1^, was as follows: isocratic from 0 to 1 min (0% B), linear gradient from 1 to 3 min (5% B), from 3 to 10 min (50% B), and 10 to 13 min (100% B), isocratic from 13 to 15 min (100% B), followed by rebalancing in the initial conditions for 5 min.

A high-resolution mass spectrometry equipment (HRMS) (MaXis 4G Q-TOF MS, Bruker Daltonics, Germany), coupled to the UHPLC system, performed the chemical mass to charge ratio (m/z) detection using an electrospray source in positive [ESI (+)—MS] and negative [ESI (–)—MS] ionization modes. Final plate offset, 500 V; capillary voltage, 3,800 V; nebulizer pressure, 4 bar; dry gas flow, 9 L min^−1^, and dry temperature, 200°C. The rate of acquisition spectra was 3.00 Hz, monitoring a mass range of 70–1,200 m/z. A sodium formate solution (10 mM HCOONa solution in 50/50 v/v isopropanol/water containing 0.2% formic acid) was injected directly through a 6-way valve at the beginning of each chromatographic run for external calibration. Ampicillin ([M+H] + *m/z* 350.11867 and [M-H]—*m/z* 348.10288) was added to each sample as an internal standard for peak normalization.

Tandem mass spectrometry (MS/MS) parameters have been adjusted to improve mass fragmentation, with collision energy ranging from 20 to 50 eV, using a step method. Precursor ions were acquired using the 3.0 s cycle time. The general AutoMS settings were: mass range, *m/z* 70–1,000 (polar fraction) and *m/z* 300–1,600 (lipidic fraction); spectrum rate, 3 Hz; ionic, positive polarity; pre-pulse storage, 8 μs; funnel 1 RF, 250.0 Vpp. The UHPLC-MS and UHPLC-MS/MS data were acquired by HyStar Application version 3.2 (BrukerDaltonics, Germany).

#### Metabolomics Data Analysis

The raw data from UHPLC-MS were exported as mzMXL files, using DataAnalysis 4.2 software (Bruker Daltonics, Germany). During data pre-processing, peak detection, retention time correction, and metabolites alignment were performed using XCMS Online (Tautenhahn et al., 2012; Gowda et al., 2014). Peak detection was performed using centWave peak detection (Δm/z = 10 ppm; minimum peak width, 5 s; maximum peak width, 20 s) and mzwid =0.015, minfrac =0.5, bw = 5 for the alignment of retention time. The unpaired parametric *t*-test (Welch *t*-test) was used for statistical analysis.

The processed data (csv file) were exported to MetaboAnalyst 5.0, and submitted to analysis in the Statistical Analysis module (Chong et al., 2019; Chong and Xia, 2020). Before the chemometric analysis, all data variables from the polar fraction were normalized by internal standard (ampicillin-rT = 7.9 min; [M+H], *m/z* = 350.1169509; sodium formate adduct-r T = 0.1 min, [M-H], *m/z* = 112.9854317), and all data variables from the lipidic fraction were also normalized by internal standard (1,2-diheptadecanoyl-sn-glycero-3-phosphocholine = 4.85 min; [M+H] + *m/z* = 762.6002192). Prior to the multivariate analysis, no data transformation was applied to the data matrices containing m/z and intensities values. All three sets of data were scaled using the Pareto scaling, which reduced the relative importance of large values, keeping data structure partially intact, and although sensitive to large fold changes, it stays closer to the original measurement than Autoscaling.

The differentially expressed peaks (DEP) were selected according to the following criteria: adjusted *P*-value (FDR) ≤ 0.050, of the Welch *t*-test. The selected DEPs were then submitted to analysis in the MS Peaks to Pathway module (Chong et al., 2019; Chong and Xia, 2020) and analyzed using the following parameters: molecular weight tolerance of 5 ppm; mixed ion mode; analysis using the mummichog algorithm (Li et al., 2013) with the default *p*-value cutoff, ranked by *p*-values, and the latest KEGG version of the *Arabidopsis thaliana* pathway library.

In the case of a DEP with two or more matched forms (isotopes) and later a matched compound with two or more DEPs, the criterion of metabolite selection applied was the mass difference comparing to the metabolite database—choosing the smallest one. Then, we used the formula and exact mass data from KEGG to perform the putative annotation of the metabolites of interest, with just one candidate on each detected ion.

The KEGG IDs of the matched compounds were then submitted to pathway analysis (integrating enrichment analysis and pathway topology analysis) and visualization in the Pathway Analysis module (Chong et al., 2019; Chong and Xia, 2020) and analyzed using the Scatter plot (testing significant features) as visualization method, the Hypergeometric Test as the enrichment method, the relative-betweenness centrality as topology analysis, and the latest KEGG version of the *A. thaliana* pathway library as reference metabolome (from October 2019).

## Metabolomics Downstream Analysis

Initially, correlation analyses were done between pairs of differentially expressed metabolites—a pairwise combination of the different scenarios evaluated—using homemade python scripts. The input data for the correlation analysis was the Log_2_(FC).

Then, the Omics Fusion web platform (Brink et al., 2016) was used to verify which pathways the metabolites selected in the correlation analysis belonged to. The input data on the platform were the KEGG IDs and the Log_2_(FC) of the metabolites and an enrichment analysis was initially carried out to discover the metabolic pathways to which they belong. The “KEGG feature distribution” module was used, which showed the metabolic pathways and the amount of metabolites differentially expressed in each pathway. To verify which were these metabolites, for each pathway, the module “Map data on the KEGG pathway” was then used.

## Results

### Gliricidia Sepium Morphophysiological Responses to Salt Stress

**Figure 1:**
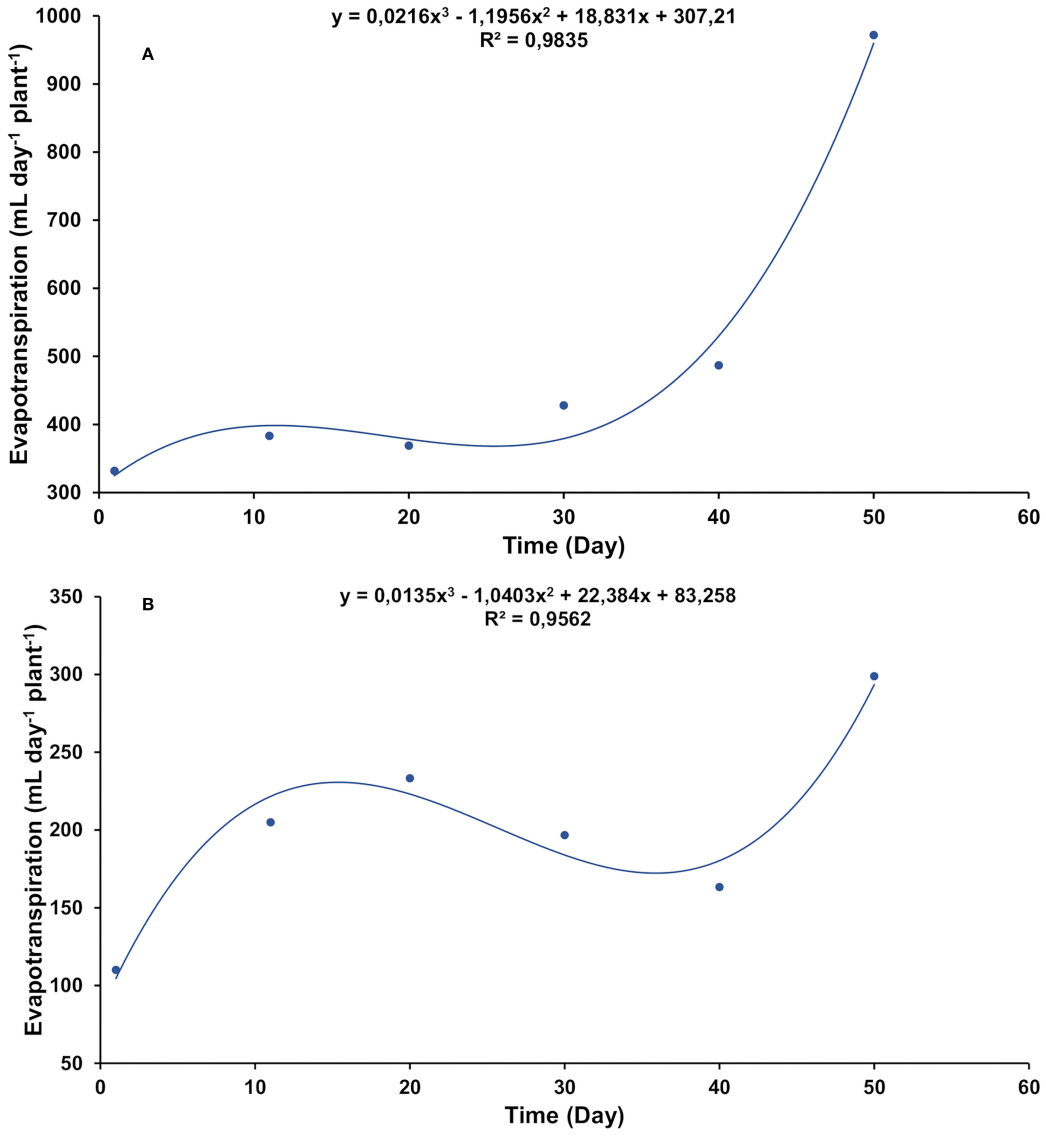
Daily evapotranspiration rate in gliricidia plants at 0.0 **(A)** and 0.8 **(B)** g of NaCl per 100 g of the substrate. The values represent the average of 11 replicates, in a timeline starting from 1 day up to 50 days of treatment.

The evapotranspiration rates of the control (Figure 1A) were higher than salt-stressed treatment (Figure 1B) throughout the experiment. However, the shape of the curve was similar in both treatments. In addition to the temporal fluctuation in transpiration rates, it was observed that as the plants got older, more water was demanded.

**Figure 2:**
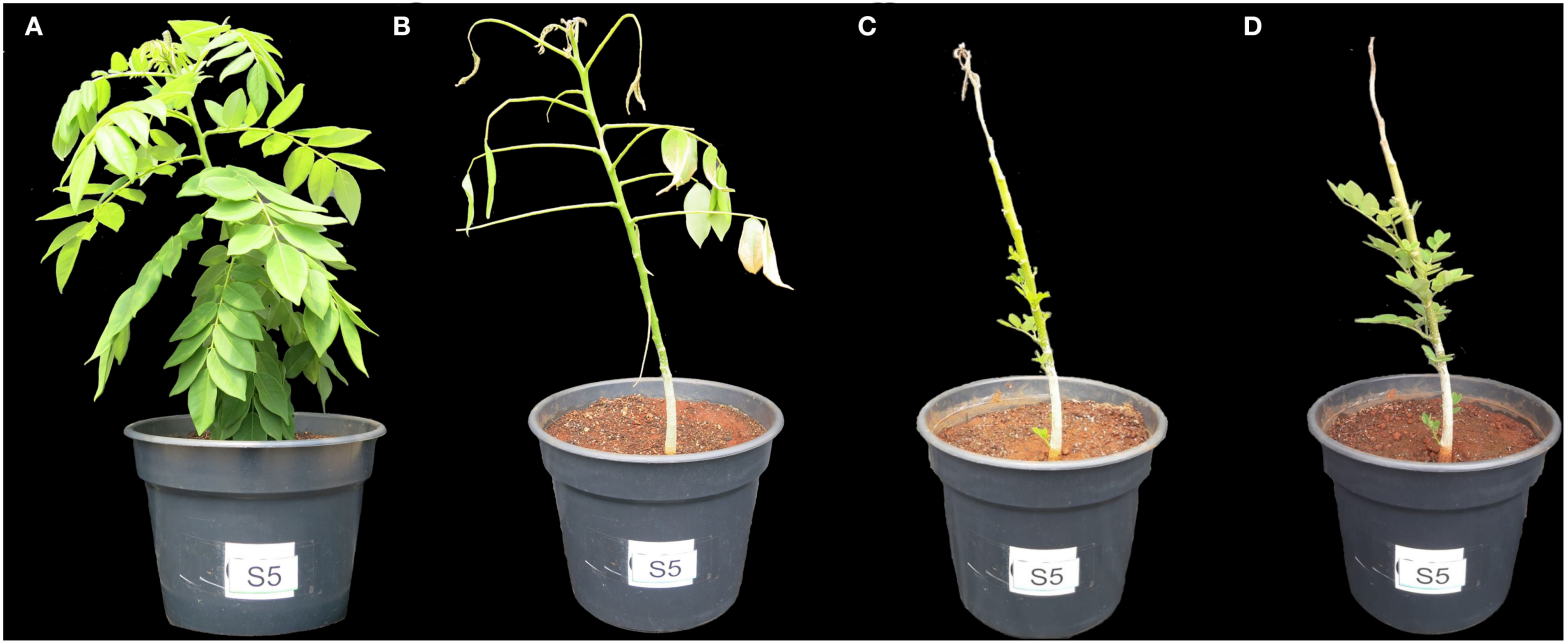
Timeline of the adaptation response in gliricidia plant under salinity stress. Plants at 0.8 g of NaCl per 100 g of the substrate. After 1 **(A)**, 5 **(B)**, 23 **(C)**, and 34 **(D)** days of stress.

The young gliricidia plants submitted to a very high level of salinity in this study showed the adaptation response previously reported by Carvalho da Silva et al. (2021). Immediately after adding salt to the substrate, the gliricidia plants were fully leafy, with completely green leaves (Figure 2A), but went on to lose all leaves before the end of the first week under salt stress (Figure 2B). Approximately 3 weeks after the onset of stress, new leaves started to emerge and continued to emerge and grow in the following weeks (Figures 2C,D).

The addition of NaCl to the substrate led to a significant increase in the concentration of exchangeable Na^+^, Mg^2+^, and K^+^. As a consequence, there was an increase in the sum of bases, cation exchange capacity, base saturation, and sodium saturation index. Additionally, a significant increase in organic matter and C was observed (Supplementary Table 1). The plants under salt stress accumulated significantly more Na^+^ and Cl^−^ than control in both the canopy and the roots. P, Cu, and Zn ions were significantly increased in the canopy under salt stress, while Fe was significantly increased in the roots.

### Gliricidia Metabolome Under Salinity Stress

Before submitting the data for analysis in the statistics module of MetaboAnalyst 5.0, the data got separated into leaf and root data and organized as follows: all in treatments dataset (control and stressed plants at 2 and 55 days after treatment—DAT); age effect—AE (samples from the control plants at 2 and 55 DAT); short-term stress—STS (the control and the stressed plants at 2 DAT); long-term stress 1—LTS1 (control and stressed plants at 55 DAT); and long-term stress 2—LTS2 (the stressed plants at 2 and 55 DAT). Each dataset had five biological replicates per treatment.

#### Leaves

Partial least squares discriminant analysis (PLS-DA) permutation tests were performed using the leaves all treatment data set to validate the model, applying permutation number = 2,000. PLS-DA is an important and widespread classification model for untargeted metabolomics, where model tendencies are described based on group separation. Groups and samples behavior can be studied through the insights given by PLS-DA on outliers, intragroup separation tendencies, and explained variance used, which add the visualization aspect to the metabolomics model. When evaluated by group separation distance, the probability that the model was created by chance was 0.06 for the lipidic-positive fraction and <0.0005 for the other two fractions (Supplementary Figure 1).

The ANOVA analysis using the leaves all treatments data set generated 4,464 peaks with a *p*-value cutoff of 8.0E-5 that were submitted to functional interpretation *via* analysis in the MS Peaks to Pathway module. After applying the initial criteria of metabolite selection, 367 peaks with a hit to just one known compound were submitted to the pathway topology analysis module, resulting in 13 pathways with a Raw *p* ≤ 0.05; they were: ubiquinone and other terpenoid-quinone biosynthesis; arginine biosynthesis; lysine biosynthesis; phenylpropanoid biosynthesis; monobactam biosynthesis; steroid biosynthesis; carotenoid biosynthesis; flavone and flavonol biosynthesis; tyrosine metabolism; valine, leucine, and isoleucine biosynthesis; porphyrin and chlorophyll metabolism, and Indole alkaloid biosynthesis. These pathways came out with 19, 11, 7, 20, 6, 19, 18, 6, 8, 10, 18, and 3 differentially expressed metabolites with the highest level of significance within the set of matched metabolites submitted to analysis, respectively (data not shown).

The changes in the metabolic profile due to the age effect, once there is a 53-day gap between the two assessments, were measured using the AE data set. The AE data set from leaves contained 816, 550, and 4,010 peaks, respectively, in the polar-positive, polar-negative, and lipidic-positive fractions; and a total of 3,168 (58.93%) peaks out of the 5,376 seen in the three fractions were differentially expressed—a differentially expressed peak (DEP) is a peak with an adjusted *P*-value (FDR) ≤ 0.05, and Log_2_ (FC) > 0 (up-regulated) or Log_2_ (FC) < 0 (down-regulated) (Table 1).

**Table 1:** Differentially expressed peaks and features in the leaves and roots of gliricidia plants submitted to salinity stress in four distinct scenarios: age effect—AE (control plants at 2 and 55 days under salinity stress—DAT); short-term stress—STS (control and the stress plants at 2 DAT); long-term stress 1—LTS1 (control and stressed plants at 55 DAT); and long-term stress 2—LTS2 (stressed plants at 2 and 55 DAT).

	**# of peaks**	**Up**	**Down**	**Non-DE**
**Leaves**				
Age effect	Control plants at 55 DAT vs. control plants at 02 DAT			
Polar-positive	816	29	483	304
Polar-negative	550	8	346	196
Lipidic-positive	4,010	1,629	673	1,708
Total	5,376	1,666	1,502	2,208
Short-term stress	Stressed plants at 02 DAT vs. control plants at 02 DAT			
Polar-positive	829	70	185	574
Polar-negative	589	3	472	114
Lipidic-positive	4,010	203	617	3,190
Total	5,428	276	1,274	3,878
Long-term stress 1	Stressed plants at 55 DAT vs. control plants at 55 DAT			
Polar-positive	788	186	97	505
Polar-negative	580	8	516	56
Lipidic-positive	4,001	75	403	3,523
Total	5,369	269	1,016	4,084
Long-term stress 2	Stressed plants at 55 DAT vs. stressed plants at 02 DAT			
Polar-positive	827	99	313	415
Polar-negative	573	36	181	356
Lipidic-positive	4,010	1,393	593	2,024
Total	5,410	1,528	1,087	2,795
**Roots**				
Age effect	Control plants at 55 DAT vs. control plants at 02 DAT			
Polar-positive	636	49	356	231
Polar-negative	392	22	78	292
Lipidic-positive	3,976	1,570	513	1,893
Total	5,004	1,641	947	2,416
Short-term stress	Stressed plants at 02 DAT vs. control plants at 02 DAT			
Polar-positive	666	104	6	556
Polar-negative	429	36	216	177
Lipidic-positive	3,993	0	28	3,965
Total	5,088	140	250	4.698
Long-term stress 1	Stressed plants at 55 DAT vs. control plants at 55 DAT			
Polar-positive	561	0	0	561
Polar-negative	380	12	148	220
Lipidic-positive	3,748	0	0	3,748
Total	4,689	12	148	4,529
Long-term stress 2	Stressed plants at 55 DAT vs. stressed plants at 02 DAT			
Polar-positive	671	37	342	292
Polar-negative	421	14	124	283
Lipidic-positive	3,990	1,992	546	1,452
Total	5,082	2,043	1,012	2,027

*The differentially expressed peaks are those with an adjusted P-value (FDR) ≤ 0.05, of the Welch t-test; and Log_2_ (Fold Change) ≠ 1*.

The short-term stress dataset from leaves was employed to evaluate how distinct are the metabolome profiles of the control and stressed plants at 2 DAT, just 1 day before the leaves started to wilt. The samples applied to evaluate the STS scenario in the leaves contained 829, 589, and 4,010 peaks, respectively, and a total of 1,550 (28.56%) peaks out of the 5,428 seen in the three fractions were differentially expressed (Table 1).

The LTS1 dataset from leaves was employed to evaluate how distinct are the metabolome profiles of the control and stressed plants at 55 DAT. The samples applied contained 788, 580, and 4,001 peaks, respectively, and a total of 1,285 (23.93%) peaks out of the 5,369 seen in the three fractions were differentially expressed (Table 1). The LTS2 dataset from leaves was employed to evaluate how distinct are the metabolome profiles of the stressed plants at two and 55 DAT. The samples applied contained 827, 573, and 4,010 peaks, respectively, and a total of 2,615 (48.34%) peaks out of the 5,410 seen in the three fractions were differentially expressed (Table 1).

**Figure 3:**
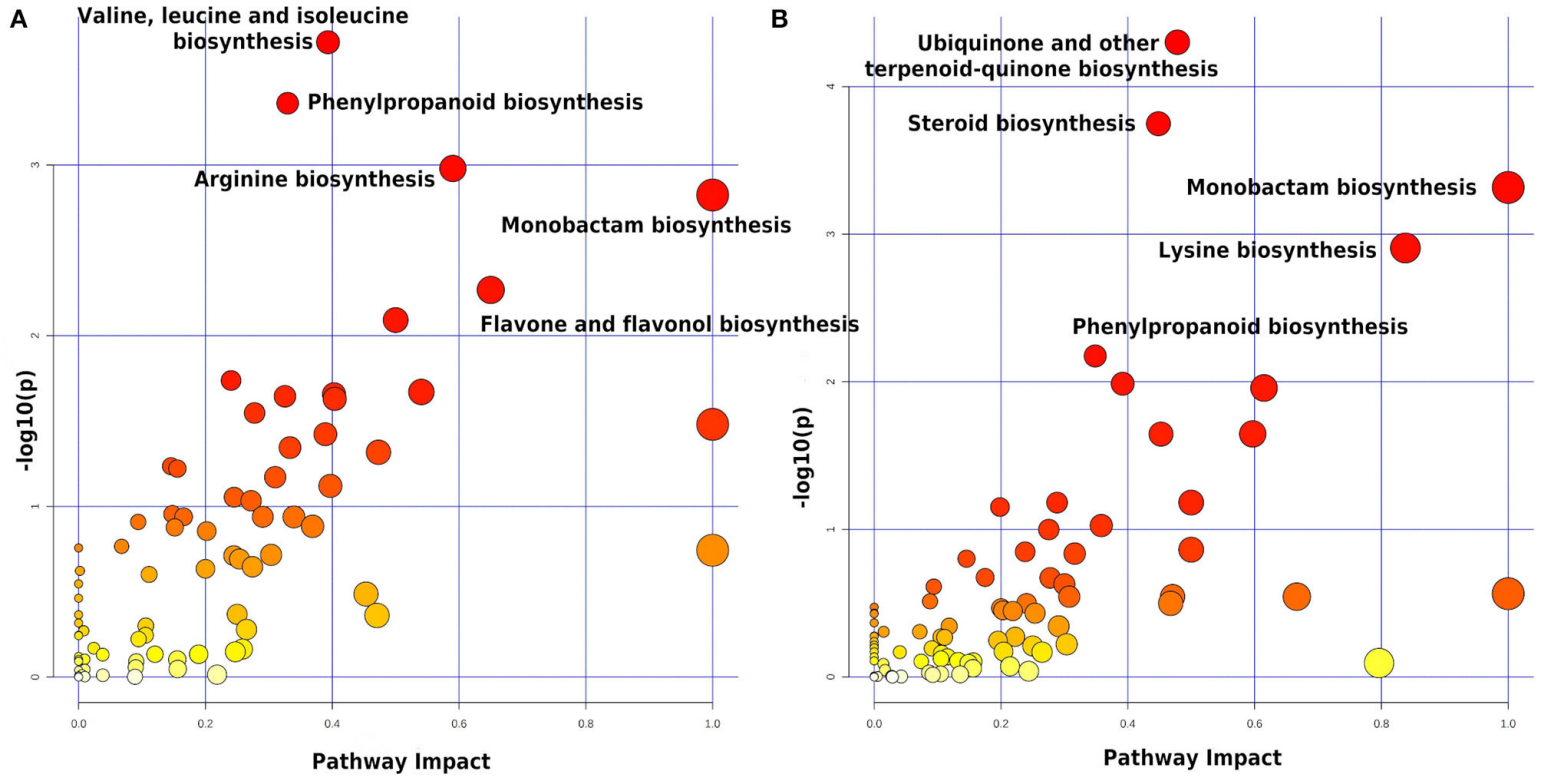
Summary of the pathway analysis of the data from leaves—STS **(A)** and LTS2 **(B)**—using the Pathway Topology Analysis modules of MetaboAnalyst 5.0. The metabolome view resulted from the analysis in the Pathway Topology Analysis module using the Hypergeometric test, the relative betweenness centrality node importance measure, and the latest KEGG version of the *A. thaliana* pathway library. STS, Short-term stress (the control and the stressed plants at 2 DAT); LTS2, Long-term stress 2 (the stressed plants at 2 and 55 DAT). Pathway impact takes into account both node centrality parameters—betweenness centrality and degree centrality—and represents the importance of annotated compounds in a specific pathway.

All 1,550 peaks differentially expressed in the STS were submitted to functional interpretation *via* analysis in the MS Peaks to Pathway module, with a *p*-value cutoff of 1.0E-5. After applying the initial criteria of metabolite selection, 197 DEPs with a hit to just one known compound were submitted to the pathway topology analysis module, resulting in 16 pathways with a raw *p* ≤ 0.05; they were: valine, leucine, and isoleucine biosynthesis; phenylpropanoid biosynthesis; arginine biosynthesis; monobactam biosynthesis; flavone and flavonol biosynthesis; indole alkaloid biosynthesis; pyruvate metabolism; lysine biosynthesis; glycine, serine, and threonine metabolism; beta-alanine metabolism; pantothenate and CoA biosynthesis; glyoxylate and dicarboxylate metabolism; C5-branched dibasic acid metabolism; fructose and mannose metabolism, anthocyanin biosynthesis, and tyrosine metabolism (Figure 3A). These pathways came out with 10, 15, 8, 5, 5, 3, 7, 4, 9, 6, 7, 8, 3, 6, 4, and 5 differentially expressed metabolites with the highest level of significance within the set of matched metabolites submitted to analysis, respectively (data not shown).

The 2,615 peaks differentially expressed in the LTS2 were also submitted to functional interpretation *via* analysis in the MS Peaks to Pathway module, with a *p*-value cutoff of 1.0 E-5. After applying the initial criteria of metabolite selection, 258 DEPs with a hit to just one known compound were submitted to the pathway topology analysis module, resulting in nine pathways with a raw *p* ≤ 0.05; they were: ubiquinone and other terpenoid-quinone biosynthesis; steroid biosynthesis; monobactam biosynthesis; lysine biosynthesis; phenylpropanoid biosynthesis; porphyrin and chlorophyll metabolism; tyrosine metabolism; arginine biosynthesis; and brassinosteroid biosynthesis (Figure 3B). These pathways came out with 17, 18, 6, 6, 15, 15, 7, 7, and 9 differentially expressed metabolites with the highest level of significance within the set of matched metabolites submitted to analysis, respectively (data not shown).

#### Roots

Partial least squares discriminant analysis permutation tests were performed using the roots all treatments data set (control and stressed plants at 2 and 55 DAT) to validate the model, applying permutation number = 2,000. When evaluated by group separation distance, the probability that the model was created by chance was 0.011 (polar-positive), 0.0005 (polar-negative), and 0.0415 (lipidic-positive) (Supplementary Figure 1).

The ANOVA analysis using the roots all in treatments dataset generated 3,600 peaks with a *p* ≤ 0.05, submitted to functional interpretation *via* analysis in the MS Peaks to Pathway module, with a *p*-value cutoff of 1.0E-5. After applying the initial criteria of metabolite selection, 326 peaks with a hit to just one known compound were submitted to the pathway topology analysis module, resulting in 10 pathways with a raw *p* ≤ 0.05; they were: steroid biosynthesis; lysine biosynthesis, ubiquinone, and other terpenoid-quinone biosynthesis; arginine biosynthesis; monobactam biosynthesis; carotenoid biosynthesis; valine, leucine, and isoleucine biosynthesis; tyrosine metabolism; phenylpropanoid biosynthesis; and porphyrin and chlorophyll metabolism. These pathways came out with 20, 7, 17, 10, 6, 18, 10, 8, 16, and 16 differentially expressed metabolites with the highest level of significance within the set of matched metabolites submitted to analysis, respectively (data not shown).

The age effect data set from roots was employed to evaluate how distinct are the metabolome profiles of the control plants at 2 and 55 DAT, a 53-day gap between the two assessments. The AE data set contained 636, 392, and 3,976 peaks, respectively, in the polar-positive, polar-negative, and lipidic-positive fractions; and a total of 2,588 (51.72%) peaks out of the 5,004 seen in the three fractions were differentially expressed (Table 1).

The STS dataset from roots was employed to evaluate how distinct are the metabolome profiles of the control and stressed plants at 2 DAT, just 1 day before the leaves started to wilt. The samples applied to evaluate the STS scenario in the leaves contained 666, 429, and 3,993 peaks, respectively, and a total of 390 (7.67%) peaks out of the 5,088 seen in the three fractions were differentially expressed (Table 1).

**Figure 4:**
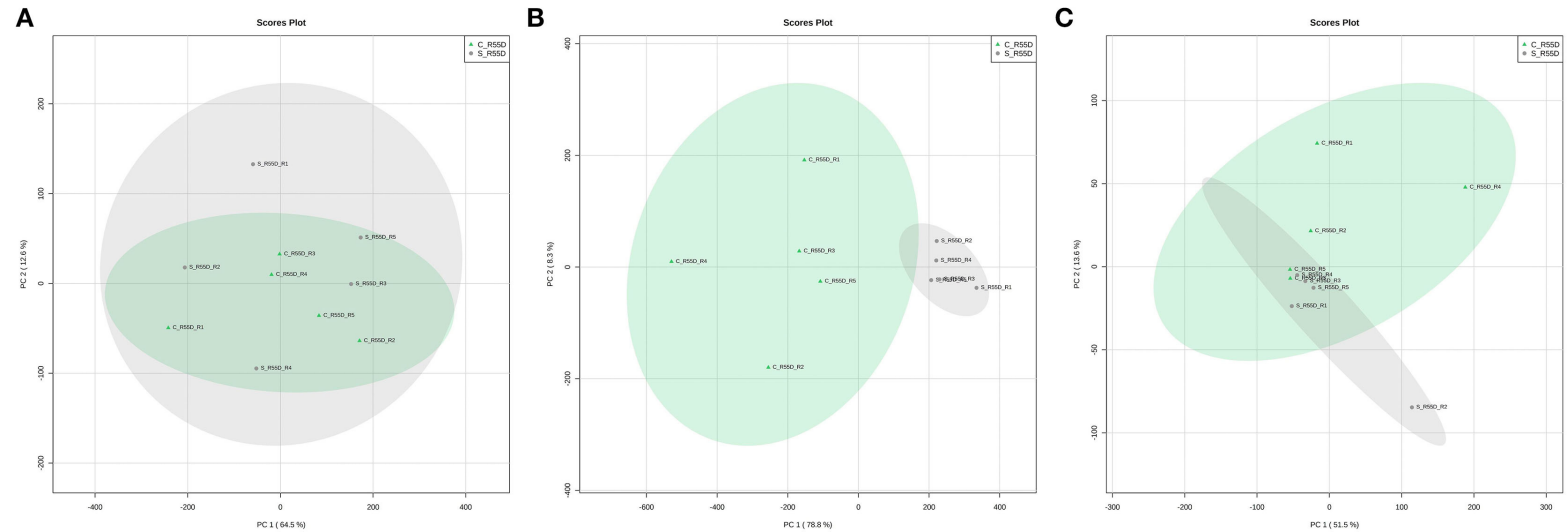
PCA score plots comparing roots samples from control and stressed plants at 55 DAT—LTS1 (Long-term stress 1). Polar positive **(A)**, polar negative **(B)**, and lipidic positive **(C)** fractions.

The LTS1 data set from roots was employed to evaluate how distinct are the metabolome profiles of the control and stressed plants at 55 DAT. The samples applied contained 561, 380, and 3,748 peaks, respectively, and a total of 160 (3.41%) peaks out of the 4,689 seen in the three fractions were differentially expressed (Table 1). A principal component analysis (PCA), an unsupervised method commonly used to identify patterns between multivariate samples, was applied to detect any inherent patterns within the data in the LTS1, and it was not able to completely separate the groups between the control and the stressed samples in the polar positive and lipidic positive fractions (Figure 4). The LTS2 data set from roots was employed to evaluate differences in the profiles from the stressed plants at two and 55 DAT. The samples applied contained 671, 421, and 3,990 peaks, respectively, and a total of 3,055 (60.11%) peaks out of the 5,082 seen in the three fractions were differentially expressed (Table 1).

All 390 peaks differentially expressed in the STS were submitted to functional interpretation *via* analysis in the MS Peaks to Pathway module, with a *p*-value cutoff of 1.0E-5. After applying the initial criteria of metabolite selection, 83 DEPs with a hit to just one known compound were submitted to the pathway topology analysis module, resulting in eight pathways with a raw *p* ≤ 0.05. The indicated pathways were: galactose metabolism; valine, leucine, and isoleucine biosynthesis; glucosinolate biosynthesis; glycine, serine, and threonine metabolism; pantothenate and CoA biosynthesis; arginine and proline metabolism; C5-branched dibasic acid metabolism; and biosynthesis of secondary metabolites—other antibiotics. These pathways came out with 6, 5, 9, 5, 6, 4, 5, 2, and 2 differentially expressed metabolites with the highest level of significance within the set of matched metabolites submitted to analysis, respectively (data not shown).

All 160 peaks differentially expressed in the LTS1 were submitted to functional interpretation *via* analysis in the MS Peaks to Pathway module, with a *p*-value cutoff of 1.0E-5. After applying the initial criteria of metabolite selection, 39 DEPs with a hit to just one known compound were submitted to the pathway topology analysis module, resulting in seven pathways with a raw *p* ≤ 0.05; they were: glyoxylate and dicarboxylate metabolism; biosynthesis of secondary metabolites—other antibiotics; beta-alanine metabolism; citrate cycle (TCA cycle); monobactam biosynthesis; alanine, aspartate, and glutamate metabolism; and pantothenate and CoA biosynthesis. These pathways came out with 5, 2, 3, 3, 2, 3, and 3 differentially expressed metabolites with the highest level of significance within the set of matched metabolites submitted to analysis, respectively (data not shown).

At last, all 3,055 peaks differentially expressed in the LTS2 were submitted to functional interpretation *via* analysis in the MS Peaks to Pathway module, with a *p*-value cutoff of 1.0E-5. After applying the initial criteria of metabolite selection, 262 DEPs with a hit to just one known compound were submitted to the pathway topology analysis module, resulting in 10 pathways with a raw *p* ≤ 0.05; they were: steroid biosynthesis; lysine biosynthesis; carotenoid biosynthesis; valine, leucine, and isoleucine biosynthesis; ubiquinone and other terpenoid-quinone biosynthesis; monobactam biosynthesis; arginine biosynthesis; phenylpropanoid biosynthesis; porphyrin and chlorophyll metabolism; and glyoxylate and dicarboxylate metabolism. These pathways came out with 20, 7, 16, 10, 14, 5, 8, 15, 14, and 9 differentially expressed metabolites with the highest level of significance within the set of matched metabolites submitted to analysis, respectively (data not shown).

### Salt Effect in Metabolites Differentially Expressed Contributing to the Adaptation Response

As the goal of this study was to look for metabolites whose behavior could give insights into the salt resistance mechanisms behind the adaptation response, both in the leaves and the roots, a series of filters were applied to select peaks differentially expressed before undergoing analysis in the MS Peaks to Pathway module of the MetaboAnalyst 5.0.

First, the 5,354 peaks in the leaf samples common to the AE, STS, and LTS2 scenarios were selected. After that, the combined effect of the salt in the short and long-term stress—denominated LTS_Final—was calculated using the following equation: FC_LTS_Final = (FC_STS ^*^ FC_LTS2 ^*^ (1/FC_AE)); the log2(FC_LTS_Final) was then subsequently calculated. After removing those peaks non-differentially expressed in the AE and the LTS_Final scenarios, the remaining 4,126 ones underwent analysis in the MS Peaks to Pathway module. A total of 353 differentially expressed peaks with a single matched compound were submitted to the Pathway Topology Analysis module (Supplementary Table 2), resulting in 12 pathways with a raw *p* ≤ 0.05; they were: ubiquinone and other terpenoid-quinone biosynthesis; phenylpropanoid biosynthesis; arginine biosynthesis; lysine biosynthesis; monobactam biosynthesis; steroid biosynthesis; flavone and flavonol biosynthesis; tyrosine metabolism; valine, leucine, and isoleucine biosynthesis; porphyrin and chlorophyll metabolism; indole alkaloid biosynthesis; and pyruvate metabolism. These pathways came out with 19, 21, 11, 7, 6, 19, 6, 8, 10, 17, 3, and 9 differentially expressed metabolites with the highest level of significance within the set of matched metabolites submitted to analysis, respectively (data not shown).

At last, there were 4,954 peaks in the root samples that were common to the AE, STS, and LTS2 scenarios. After submitting them to the same treatment mentioned above, a group of 3,557 peaks was left for analysis in the MS Peaks to Pathway module. A total of 309 differentially expressed peaks with a single matched compound were submitted to the Pathway Topology Analysis module (Supplementary Table 2), resulting in 11 pathways with a raw *p* ≤ 0.05; they were: steroid biosynthesis; lysine biosynthesis; monobactam biosynthesis; ubiquinone and other terpenoid-quinone biosynthesis; arginine biosynthesis; valine, leucine, and isoleucine biosynthesis; carotenoid biosynthesis; phenylpropanoid biosynthesis; alanine, aspartate, and glutamate metabolism; porphyrin and chlorophyll metabolism; and tyrosine metabolism. These pathways came out with 20, 7, 6, 16, 9, 10, 16, 16, 9, 16, and 7 differentially expressed metabolites with the highest level of significance within the set of matched metabolites submitted to analysis, respectively (data not shown).

## Discussion

One of the manners by which salt affects plants is through the osmotic effect. Salt reduces the osmotic potential and, consequently, diminishes the soil water potential, making it difficult for plants to absorb water (Sánchez-Blanco et al., 2004; Franco et al., 2011; Hanin et al., 2016). Under such conditions, there is a reduction in the stomatal opening (Acosta-Motos et al., 2017). As a result, the plants transpire less, and therefore less water is lost by evaporation, as occurred with the salt-stressed gliricidia plants. However, irrespective of salt stress, weather variables, such as radiation, temperature, and relative humidity, continue to exert their effects on evapotranspiration. For this, the shape of both evapotranspiration curves, stressed or unstressed by salt, was the same (Figure 1).

The gliricidia control plants exerted their maximum transpiration capacity, as there was no obstacle in the soil limiting water availability. They had full foliage and high rates of evapotranspiration (Figures 1A, 2A). In the salt-stressed gliricidia plants, the osmotic shock caused by the abrupt addition of salt to the substrate led to severe defoliation (Figure 2B). This fact is a drastic artifice used by plants to reduce the transpiring surface and maintain water status (Alarcón et al., 2006), although it could also be a mechanism of salt exclusion (Acosta-Motos et al., 2017). The fact is that, after metabolic adjustment to that stressful situation, gliricidia plants developed new leaves and continue their development (Figures 2C,D).

The availability of exchangeable Na in the substrate represented 36% of the base saturation and was at least six times higher than K in the salt-stressed treatments. Even under these conditions, the gliricidia plants only absorbed a tiny part of the Na, while they absorbed Mg, Ca, and K just like the control ones (Supplementary Table 1), which suggests that it limited the entry of Na by the roots (Hanin et al., 2016) and maintain the nutritional balance. Therefore, it is a typical salt-excluding plant (Acosta-Motos et al., 2017).

**Figure 5:**
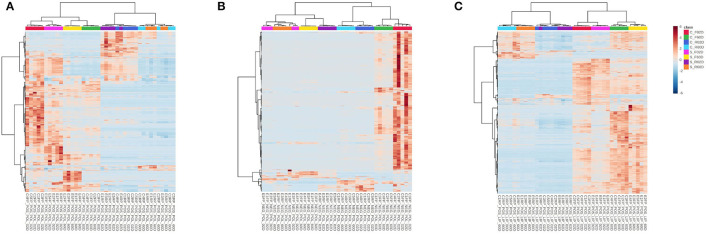
Heatmap analysis of the all treatments data sets (control and stressed plants at 2 and 55 DAT). Blue color indicates low intensity and red color indicates high intensity. The upper row represents sample groups (C—Control, S—Stressed, F—Leaves, and R—Roots). Polar positive **(A)**, polar negative **(B)**, and lipidic positive **(C)** fractions.

The heatmaps generated out of the metabolome from the roots of young gliricidia plants submitted to salinity stress, using the all treatments datasets, revealed much closer proximity between treatments within the short-term scenario, as well as within the long-term one, but only for the polar positive and lipidic positive fractions evaluated (Figure 5). The hierarchical clustering heatmaps were generated in MetaboAnalyst 5.0 using normalized data as data source, autoscale features as standardization, Pearson as distance measure, and Ward as a clustering method. To a certain extent, the same was true for the metabolome from the leaves (Figure 5). That led us to postulate that, once adapted to high salinity stress, most likely *via* a salt-excluding mechanism that limited the entry of Na in the roots, gliricidia plants resumed growth and went back to have almost the same metabolome profile as the control plants at the end of the experiment.

In the leaves, the phenylpropanoid biosynthesis pathway always appeared as one of the most affected pathways, independent of the strategy used to analyze the different data sets, with 21 metabolites differently expressed. When further evaluating the changes experienced by these metabolites in short-term and long-term stress, we found that 14 did downregulate and seven upregulate at 55 DAT in the stressed plants, in comparison with the control plants also at 55 DAT. Besides that, 12 were downregulated in the short-term but not long-term, two differentially regulated at short-term and long-term, and seven differentially regulated only at long-term (Table 2).

**Table 2:** Differentially expressed peaks and features from the phenylpropanoid biosynthesis pathway (leaves), steroid biosynthesis (roots), and lysine biosynthesis (roots), in gliricidia plants submitted to salinity stress in three distinct scenarios: age effect—AE (control plants at 2 and 55 days under salinity stress—DAT), short-term stress—STS (control and the stress plants at 2 DAT); and long-term stress 2—LTS2 (stressed plants at 2 and 55 DAT).

**Pathway/** **organ**	**m.z**	**Mode**	**Matched.** **Compound**	**Matched.** **Form**	**Mass.** **Diff**	**Fold_** **Change_** **AE**	**Log2(FC)_** **AE**	**Profile_** **AE**	**Fold_** **Change_** **STS**	**Log2(FC)_** **STS**	**Profile_** **STS**	**Fold_** **Change_** **LTS2**	**Log2(FC)_** **LTS2**	**Profile_** **LTS2**	**Fold_** **Change_** **LTS_** **Final**	**Log2(FC)_** **LTS_** **Final**	**Profile_** **LTS_** **Final**
Phenylpropanoid biosynthesis/leaf	163.0399	Negative	C05608	M-H+O[-]	1.50E-04	0.32	−1.63	Down	0.02	−5.72	Down	1.00	0.00	Non-DE	0.06	−4.08	Down
	165.0558	Negative	C12206	M-H[-]	3.44E-05	0.15	−2.75	Down	0.03	−4.92	Down	1.00	0.00	Non-DE	0.22	−2.18	Down
	179.0350	Negative	C10945	M-H+O[-]	2.75E-05	0.15	−2.71	Down	0.02	−5.72	Down	1.00	0.00	Non-DE	0.12	−3.01	Down
	223.0611	Negative	C05610	M-H+O[-]	9.97E-05	0.24	−2.06	Down	0.08	−3.74	Down	1.00	0.00	Non-DE	0.31	−1.67	Down
	250.0721	Negative	C05619	M+ACN-H[-]	8.96E-06	0.03	−5.24	Down	0.01	−6.51	Down	1.00	0.00	Non-DE	0.42	−1.27	Down
	309.0965	Positive	C10434	M-CO+H[1+]	2.82E-04	0.52	−0.94	Down	0.32	−1.66	Down	1.00	0.00	Non-DE	0.61	−0.72	Down
	338.0965	Negative	C12208	M(C13)-H[-]	2.10E-04	0.08	−3.63	Down	0.01	−6.97	Down	1.00	0.00	Non-DE	0.10	−3.33	Down
	341.0878	Negative	C05839	M-H+O[-]	4.68E-05	0.05	−4.25	Down	0.02	−5.72	Down	1.00	0.00	Non-DE	0.36	−1.46	Down
	401.1457	Negative	C00761	M+CH3COO[-]	8.98E-04	1.00	0.00	Non-DE	0.03	−5.16	Down	1.00	0.00	Non-DE	0.03	−5.16	Down
	355.0673	Negative	C02887	M-H+O[-]	2.13E-04	1.00	0.00	Non-DE	0.05	−4.47	Down	1.00	0.00	Non-DE	0.05	−4.47	Down
	327.1088	Negative	C05855	M-H+O[-]	3.05E-04	0.13	−2.91	Down	0.03	−5.06	Down	2.76	1.47	Up	0.62	−0.68	Down
	85.52103	Positive	C00423	M+H+Na[2+]	1.80E-04	0.01	−6.15	Down	1.00	0.00	Non-DE	0.01	−6.62	Down	0.73	−0.46	Down
	632.2630	Positive	C18070	M+H[1+]	2.78E-03	1.00	0.00	Non-DE	1.00	0.00	Non-DE	0.46	−1.11	Down	0.46	−1.11	Down
	293.0632	Positive	C00482	M+HCOONa[1+]	6.87E-05	1.00	0.00	Non-DE	1.00	0.00	Non-DE	0.04	−4.48	Down	0.04	−4.48	Down
	105.0697	Positive	C00903	M-CO+H[1+]	1.34E-04	0.02	−5.87	Down	0.03	−5.32	Down	1.00	0.00	Non-DE	1.46	0.55	Up
	455.1160	Positive	C01175	M+HCOONa[1+]	3.74E-05	0.10	−3.26	Down	0.35	−1.52	Down	1.00	0.00	Non-DE	3.32	1.73	Up
	108.0387	Positive	C02947	M(C13)+3H[3+]	5.14E-04	0.31	−1.71	Down	2.20	1.14	Up	0.31	−1.70	Down	2.22	1.15	Up
	107.0488	Positive	C02666	M-C3H4O2+H[1+]	3.25E-04	0.22	−2.15	Down	1.00	0.00	Non-DE	4.60	2.20	Up	20.45	4.35	Up
	167.0701	Positive	C01494	M-CO+H[1+]	1.29E-04	0.58	−0.79	Down	1.00	0.00	Non-DE	5.26	2.39	Up	9.11	3.19	Up
	333.1531	Positive	C00933	M+Na[1+]	1.63E-03	0.60	−0.74	Down	1.00	0.00	Non-DE	0.63	−0.66	Down	1.05	0.08	Up
	180.0664	Negative	C00079	M-H+O[-]	2.50E-04	1.00	0.00	Non-DE	1.00	0.00	Non-DE	3.56	1.83	Up	3.56	1.83	Up
Steroid biosynthesis/roots	361.3266	Positive	C15780	M-H4O2+H[1+]	1.28E-03	0.15	−2.71	Down	1.00	0.00	Non-DE	0.18	−2.44	Down	1.20	0.27	Up
	435.3599	Positive	C15782	M+Na[1+]	1.42E-04	1.00	0.00	Non-DE	1.00	0.00	Non-DE	1.42	0.50	Up	1.42	0.50	Up
	397.3826	Positive	C22121	M-CO+H[1+]	1.42E-04	1.00	0.00	Non-DE	1.00	0.00	Non-DE	1.95	0.97	Up	1.95	0.97	Up
	415.3940	Positive	C01753	M+H[1+]	5.60E-04	5.83	2.54	Up	1.00	0.00	Non-DE	7.92	2.99	Up	1.36	0.44	Up
	449.3752	Positive	C01054	M+Na[1+]	2.19E-04	2.24	1.16	Up	1.00	0.00	Non-DE	3.81	1.93	Up	1.70	0.77	Up
	441.4077	Positive	C08830	M+H[1+]	1.35E-03	5.67	2.50	Up	1.00	0.00	Non-DE	9.43	3.24	Up	1.66	0.73	Up
	437.3754	Positive	C15915	M+Na[1+]	6.85E-05	2.07	1.05	Up	1.00	0.00	Non-DE	2.08	1.05	Up	1.00	0.01	Up
	397.3830	Positive	C01943	M-HCOOH+H[1+]	6.38E-05	3.64	1.86	Up	1.00	0.00	Non-DE	3.65	1.87	Up	1.00	0.01	Up
	465.3501	Positive	C11523	M+K[1+]	1.11E-03	2.14	1.10	Up	1.00	0.00	Non-DE	2.21	1.14	Up	1.03	0.04	Up
	457.3674	Positive	C22120	M+H[1+]	1.36E-04	3.96	1.99	Up	1.00	0.00	Non-DE	1.00	0.00	Non-DE	3.96	1.99	Up
	369.3516	Positive	C01189	M-H2O+H[1+]	2.37E-05	1.00	0.00	Non-DE	1.00	0.00	Non-DE	4.14	2.05	Up	4.14	2.05	Up
	479.3861	Positive	C00751	M+HCOONa[1+]	1.35E-04	5.13	2.36	Up	1.00	0.00	Non-DE	7.59	2.92	Up	1.48	0.56	Up
	489.3937	Positive	C22116	M+H2O+H[1+]	1.72E-04	6.64	2.73	Up	1.00	0.00	Non-DE	5.29	2.40	Up	0.80	−0.33	Down
	383.3671	Positive	C11508	M-CO+H[1+]	5.36E-05	3.21	1.68	Up	1.00	0.00	Non-DE	2.99	1.58	Up	0.93	−0.10	Down
	429.3728	Positive	C22123	M+H2O+H[1+]	1.05E-05	3.00	1.58	Up	1.00	0.00	Non-DE	1.79	0.84	Up	0.60	−0.74	Down
	445.3477	Positive	C22119	M(S34)+H[1+]	7.31E-05	6.47	2.69	Up	1.00	0.00	Non-DE	3.53	1.82	Up	0.55	−0.87	Down
	384.1425	Positive	C00448	M(C13)+H[1+]	7.79E-04	0.61	−0.72	Down	1.00	0.00	Non-DE	0.58	−0.79	Down	0.95	−0.07	Down
	443.3052	Positive	C05437	M+NaCl[1+]	1.03E-04	44.02	5.46	Up	1.00	0.00	Non-DE	30.00	4.91	Up	0.68	−0.55	Down
	400.3654	Positive	C15777	M(C13)+H[1+]	1.86E-04	3.63	1.86	Up	1.00	0.00	Non-DE	3.12	1.64	Up	0.86	−0.22	Down
	541.3193	Positive	C03428	M-HCOOH+H[1+]	1.45E-03	27.03	4.76	Up	1.00	0.00	Non-DE	14.04	3.81	Up	0.52	−0.95	Down
Lysine biosynthesis/roots	88.0394	Positive	C00049	M-HCOOH+H[1+]	5.11E-05	0.44	−1.18	Down	1.00	0.00	Non-DE	0.46	−1.12	Down	1.04	0.06	Up
	130.0497	Positive	C03082	M-HCOOK+H[1+]	8.97E-05	0.46	−1.11	Down	1.00	0.00	Non-DE	0.56	−0.83	Down	1.21	0.28	Up
	256.0424	Positive	C20258	M+HCOONa[1+]	3.00E-04	0.01	−6.17	Down	1.00	0.00	Non-DE	0.16	−2.65	Down	11.47	3.52	Up
	100.0393	Positive	C00441	M-H2O+H[1+]	6.94E-06	0.36	−1.49	Down	1.00	0.00	Non-DE	0.29	−1.81	Down	0.80	−0.32	Down
	144.0652	Positive	C03972	M-CO+H[1+]	1.85E-04	0.16	−2.61	Down	1.00	0.00	Non-DE	0.08	−3.57	Down	0.51	−0.96	Down
	191.1023	Positive	C00666	M+H[1+]	3.22E-04	0.13	−2.92	Down	1.00	0.00	Non-DE	0.01	−7.02	Down	0.06	−4.10	Down
	106.0384	Positive	C00680	M+H+Na[2+]	3.20E-04	0.35	−1.51	Down	1.00	0.00	Non-DE	0.29	−1.81	Down	0.81	−0.30	Down

*The differentially expressed peaks are those with an adjusted P-value (FDR) ≤ 0.05, of the Welch t-test; and Log_2_ (Fold Change) ≠ 1. LTS_Final, Effect of salt stress in the long-term discounted the age effect; Down, Downregulated; Up, Upregulated; Non-DE, Non-Differentially Expressed. If False Discovery Rate (FDR) ≤ 0.05, then Profile was called Non-DE, and FC (Fold Change) became 1.0*.

*Down, Downregulated; Up, Upregulated; Non-DE, Non-Differentially Expressed. If False Discovery Rate (FDR) ≤ 0.05, then Profile = Non-De, and FC = 1. Scenarios: age effect—AE (samples from the control plants at 2 and 55 days after treatment—DAT); short-term stress—STS (the control and the stressed plants at 2 DAT); long-term stress 1—LTS1 (the control and the stressed plants at 55 DAT); long-term stress 2—LTS2 (the stressed plants at 2 and 55 DAT); and LTS_Final = Effect of salt stress in the long-term discounted the age effect*.

Taken together, the results that are shown in this study corroborate the previous work done by Carvalho da Silva et al. (2021), who showed that this pathway is highly affected in the leaves of young gliricidia plants by salt stress, but also that most of the salt effect occurs in the short-term. However, it goes forward and shows that most of those changes remained in the long-term, and also that some metabolites change in expression only during the process of adaptation to high salinity experienced by these plants. In summary, this pathway plays a role in the response of young gliricidia plants to high salinity throughout the entire adaptation response, starting in the short term and continuing in the long one.

Zhu et al. (2021) also applied an integrated transcriptomic and metabolomic analysis to show that the Phenylpropanoid biosynthesis pathway had genes and metabolites that show a significant correlation in the roots of salt-stressed *Sophora alopecuroides*, which is a leguminous perennial herb that is an excellent sand-fixing pioneer plant distributed mainly in the desert and semi-desert areas of Northwest China.

Phenylpropanoids, a class of secondary metabolites showing indispensable roles in plant survival, are synthesized from phenylalanine (or tyrosine) through a series of enzymatic reactions (Deng and Lu, 2017). Their biosynthesis includes a collection of the first two or three steps of the phenylpropanoid pathway, which redirects carbon flow from primary metabolism to phenylpropanoid metabolism; and after the generation of intermediates in the initial steps, the carbon flow is channeled into specific branch pathways to produce flavonoids, stilbenes, monolignols, phenolic acids, and coumarins (Deng and Lu, 2017).

In the present study, L-Phenylalanine (C00079) is one of the three metabolites from this pathway that only differentiated in the long-term stress, experiencing an increase of 3.56X in its peak intensity due exclusively to the salt effect. The other two differentiated metabolites were Sinapic acid (C00482) and N1,N5,N10-Tricaffeoyl spermidine (C18070), which experienced a decrease of 96 and 54%, respectively, also due exclusively to the salt effect.

**Figure 6:**
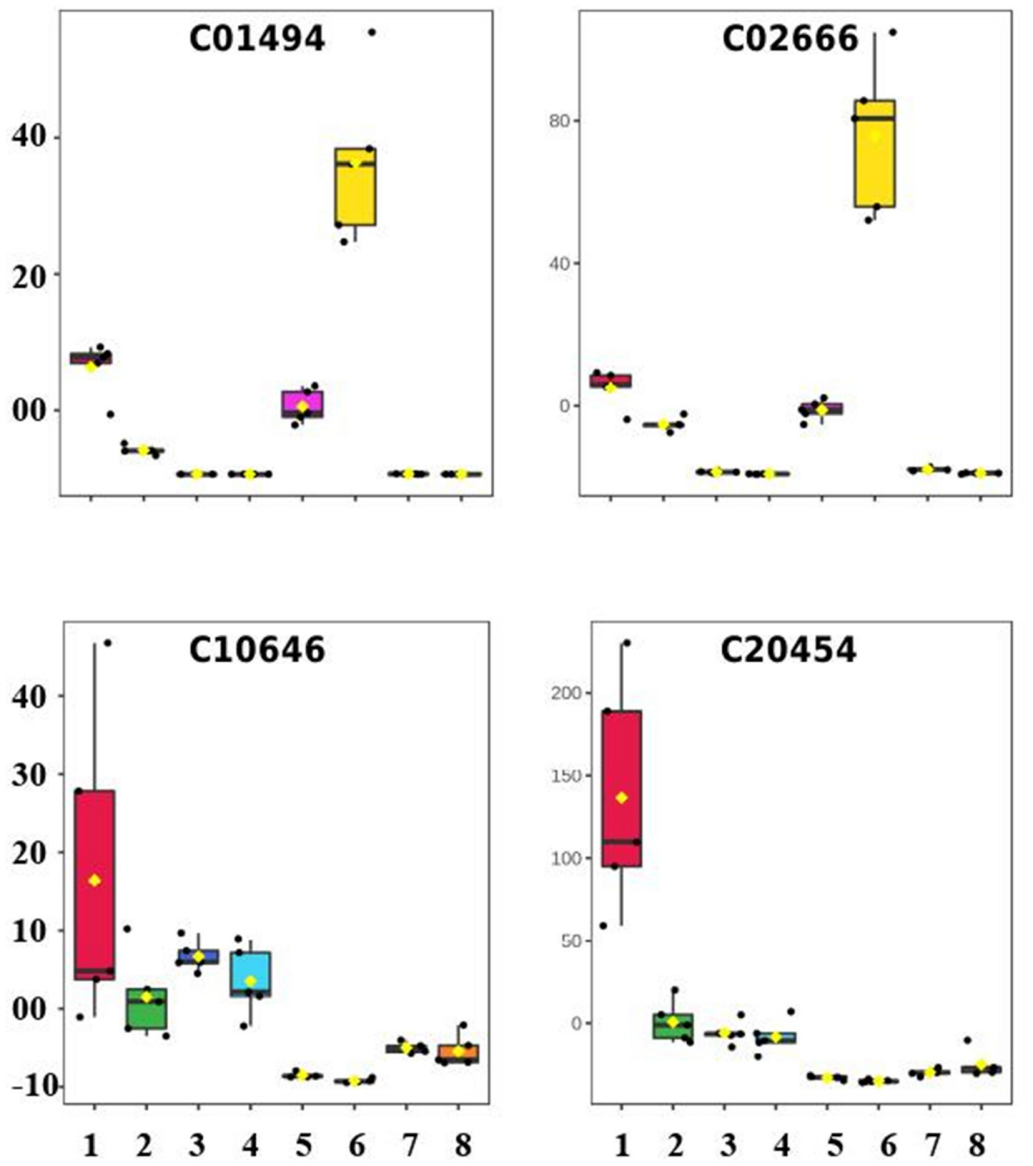
Box plot showing the normalized concentration of metabolites in the leaves and roots of young gliricidia plants submitted to salt stress. (The expression profiles of Ferulic acid (C01494), Coniferyl aldehyde (C02666) (+)-Lariciresinol (C10646), and (–)-Lariciresinol (C20454) in the following treatments: 1—C_L02D, 2—C_L55D, 3—C_R02D, 4—C_R55D, 5—S_L02D, 6—S_L55D, 7—S_R02D, 8—S_R55D, where C—Control, S—Stressed, F—Leaves, R—Roots, 02D—short-term stress, and 60D—long-term stress).

Ferulic acid (C01494) and Coniferyl aldehyde (C02666) were the two metabolites that experienced the most increase in expression level in the long-term—due exclusively to the salt effect—among those 21 metabolites differentially expressed in this pathway, experiencing 9.11X and 20.45X increase in intensity, respectively (Figure 6). Ferulic acid belongs to the hydroxycinnamic acid group, which is one of the two major subgroups of phenolic acids, likely produced by the oxidation of coniferaldehyde under the catalysis by coniferyl-aldehyde dehydrogenase. Many steps of the phenolic acid pathway are unknown, and it is still under revision (Deng and Lu, 2017). Recently, Linić et al. (2021) showed that exogenous application of a 10.0 μM solution of ferulic acid attenuated effects on salt-stressed Chinese cabbage plants, causing a decrease in proline and salicylic acid.

Besides ferulic acid and coniferyl aldehyde, three other metabolites upregulated at 55 DAT in the stressed plants, in comparison with the control plants also at 55 DAT; they were: 1-O-Sinapoyl-beta-D-glucose (C01175), Cinnamaldehyde (C00903), and 4-Coumaroylshikimate (C02947), which experienced an increase of 3.32X, 1.46X, and 2.22X, respectively (data not shown). According to Chun et al. (2019), enzymes, such as phenylalanine ammonia-lyase (PAL), cinnamoyl CoA reductase (EC 1.2.1.44), ferulate 5-hydroxylase (F5H), caffeate 3-O-methyltransferase (EC 2.1.1.68), and cinnamyl alcohol dehydrogenase (EC 1.1.1.195)—to mention a few—take part in the synthesis of monolignols. Peroxidases and laccases polymerize monolignols to yield lignin as a final product. L-Phenylalanine, Ferulic acid, Coniferyl aldehyde, and Cinnamaldehyde, which the expression level increased due to the salt effect in this present study, are directly linked to these five enzymes.

When silencing the *CcoAOMT1* gene in *A. thaliana* and demonstrating that the mutants became phenotypically hypersensitive to salt stress, Chu and colleagues provided molecular and genetic evidence indicating the importance of enhanced lignin accumulation in the cell wall of this plant species during the responses to salt stress (Chun et al., 2019). The lignin accumulation and the strong expression of lignin biosynthetic genes are factors key to acquiring salt tolerance in plants, and, based on the results from our present study, we postulate that this phenomenon is playing a role in the adaptation of young gliricidia plants to high levels of salt stress. Additional studies are necessary to investigate this hypothesis.

Only 3.4% of the peaks show differential expression in the LTS1 scenario, in contrast to the AE (51.7%) and LTS2 (60.1%) ones, in the roots of gliricidia plants. The principal component analysis (PCA) did not completely separate the control and stressed samples in the polar positive and the lipidic positive fraction (Figure 4), and all 160 differentially expressed peaks in the subsequent analysis came from the negative one. Together, these results show that the young gliricidia plants did experience a metabolic adjustment that led their metabolic status in the roots to pretty much the same one of the control plants and that this adjustment most likely took place after the loss of the leaves.

Only 39 DEPs with a hit to just one known compound were found in LTS1 and submitted to the pathway topology analysis module, resulting in seven pathways with a raw *p* ≤ 0.05. However, only the biosynthesis of secondary metabolites—other antibiotics one suffered the utmost impact seen, with two lignans downregulated in the short-term and kept at a low level in the long-term stress (+)-Lariciresinol (C10646), and (–)-Lariciresinol (C20454) (Figure 6). This finding opposes Xiao et al. (2020), who showed that the upregulation of lariciresinol biosynthesis in *Isatis indigotica*, particularly in tetraploids compared to diploids, improved root development, and enhanced salt and drought stress tolerance.

In the roots, the steroid biosynthesis is the most affected pathway in the long-term stress, with 20 differently expressed metabolites. Fourteen of them are from the phytosterol biosynthesis module (M00917—squalene 2,3-epoxide = > campesterol/sitosterol). When evaluating the changes experienced by these 20 metabolites in the short-term and long-term stress, none of them expressed differentially at 02 DAT (Table 2). Lathosterol (C01189) had the utmost change in the expression level due exclusively to salt stress, with a 4.14X increase after the short-term. Cycloeucalenone (C22121), with 1.95X increase, and Squalene 2,3-epoxide (C01054) and 24-Methylidenecycloartanol (C08830), both with approximately 1.7X.

Over 250 different plant sterols are known, among which campesterol, sitosterol, and stigmasterol are the most abundant ones in most plants (Moreau et al., 2018). An overall summary of the enzymes involved in the biosynthesis of phytosterols is lacking, and very little research on the metabolism and regulation of phytosterols biosynthesis has been reported (Zhang et al., 2020). According to Zhang et al. (2020), sterol profiles vary greatly among plant species and seem to be accumulated more in plants with higher tolerance to salinity; these changes of phytosterols accumulation can indicate plant adaptability to stresses to a certain extent. Phytosterols may help plants adapt to environmental stresses by adjusting membrane structure and characteristics, including the recruitment of specific proteins attached to the plasma membrane to transduce signals (Zhang et al., 2020). Maintenance of membrane homeostasis represents one of the principal functions of sterols in plant cells (Rogowska and Szakiel, 2020), and phytosterols and their corresponding esters have a pivotal role in imparting tolerance to abiotic stresses by maintaining cell membrane integrity (Kumar et al., 2018).

In the roots of salt-stressed gliricidia plants, the Lysine biosynthesis appeared as the second most affected pathway in the long-term, with seven metabolites expressed differently; being all of them from the DAP aminotransferase pathway module (Hudson et al., 2006). When further evaluating the changes experienced by these seven metabolites in the short-term and long-term stress, four of them were downregulated (C00441, C00666, C00680, and C03972) and three upregulated (C00049, C03082, and C20258) in the long-term, due exclusively to salt stress. None of them expressed differentially at 02 DAT, short-term (Table 2).

In higher plants, the synthesis of Lysine (Lys) occurs in plastids *via* the diaminopimelate (DAP) pathway, and no evidence exists for its cytosolic synthesis (Hudson et al., 2006; Kishor et al., 2020). When accumulated in high concentrations, Lys may be toxic to the plants and must then undergo degradation *via* the cadaverine, the saccharopine, or the NHP pathway (Arruda and Barreto, 2020; Kishor et al., 2020). In the present study, Lys was not among the metabolites expressed differentially in the roots of gliricidia plants due to salt stress; so, it is not possible to say whether these plants are experiencing energy limitations or not. However, as six metabolites (C00026, C00042, C00164, C00408, C00449, and C04076) from the Lys degradation pathways were found in the roots of gliricidia plants after 55 days under stress, and all of them at levels similar to the ones in the control plants (data not shown), one can postulate that Lys degradation did not play a role in promoting the adaptation response.

The (2S,4S)-4-Hydroxy-2,3,4,5-tetrahydrodipicolinate—HTPA (C20258) metabolite experienced, by far, the utmost increase (11.47X) in expression level in the long-term after removing the age effect; and the LL-2,6-Diaminopimelic acid (C00666) the utmost decrease (94%). Since HTPA expression upregulates due to salt stress, one can infer that the protein expressed by a putative homolog of the *dapA* gene in *G. sepium* is Lys-insensitive or that the amount of Lys is not enough to trigger a feedback inhibition (Galili et al., 2001). The 4-hydroxy-tetrahydrodipicolinate synthase (EC 4.3.3.7), which catalyzes the production of HTPA, is coded by the *dapA* gene (Soares da Costa et al., 2021).

## Conclusion

In the salt-stressed gliricidia plants, the osmotic shock caused by the abrupt addition of salt to the substrate led to severe defoliation. After a metabolic adjustment to that stressful situation, those plants released new leaves and continued their development. These salt-adapted gliricidia plants only absorbed a tiny part of the Na present in the substrate while absorbing Mg, Ca, and K just like the control ones. The limited entry of Na and the maintenance of the nutritional balance in the roots, plus the fact that the roots metabolome profiles of the control and adapted plants were very similar, led us to postulate that these plants are salt-excluding plants that are adapted to high salinity stress *via* two salt-excluding mechanisms, starting in the canopy—severe defoliation—and concluding in the roots—limited entry of Na.

This present study not only corroborates our previous study that indicated that the phenylpropanoid biosynthesis pathway has a role in the response of gliricidia plants to a very high level of salinity (Carvalho da Silva et al., 2021) but went forward and also showed that most of the changes experienced by this pathway in the leaves during the short-term stress, remained in the long-term. However, some metabolites from this pathway play a role only in the long-term response to this stress. In summary, this pathway plays a role throughout the entire adaptation process, starting in the short term and continuing in the long.

Based on the results from our present study—from leaves and roots—we can also postulate that the accumulation of lignin and some phytosterols, as well as lysine biosynthesis—but not degradation, play a role in promoting the adaptation response of gliricidia plants to a very high level of salinity. However, additional studies are necessary to investigate this hypothesis.

## Data Availability Statement

The data-sets used and/or analyzed during the current study are available from the corresponding author on reasonable request.

## Author Contributions

PA, CS, and MS designed the study. ÍB, TC, VB, JRo, and JRi performed the experiments and generated the data. ÍB and MS wrote first draft of the manuscript, which was extensively edited and approved the submitted version by all authors. MS was responsible for the funding acquisition, project administration, and group supervision. All authors contributed to the article and approved the submitted version.

## Funding

The grant (01.13.0315.00—DendePalm Project) for this study was awarded by the Brazilian Ministry of Science, Technology, and Innovation (MCTI) *via* the Brazilian Research and Innovation Agency (FINEP). The authors confirm that the funder had no influence over the study design, the content of the article, or the selection of this journal.

## Conflict of Interest

The authors declare that the research was conducted in the absence of any commercial or financial relationships that could be construed as a potential conflict of interest.

## Publisher's Note

All claims expressed in this article are solely those of the authors and do not necessarily represent those of their affiliated organizations, or those of the publisher, the editors and the reviewers. Any product that may be evaluated in this article, or claim that may be made by its manufacturer, is not guaranteed or endorsed by the publisher.

## Supplementary Material

Supplementary Figure 1 PLS-DA permutation validation evaluated by group separation distance, applying permutation number = 2,000. From the all treatments leaves **(A,C,E)** and roots **(B,D,F)** data sets (control and stressed plants at 2 and 55 DAT). Polar-positive **(A,B)**, polar-negative **(C,D)**, and lipidic-positive **(E,F)** fractions.

Supplementary Table 1 Physicochemical properties of the substrate and the mineral composition of canopy and roots of gliricidia plants at the end of the experiment, according to the NaCl level (g of NaCl per 100 g of substrate) applied. The values represent an average of five samples, followed by the standard deviation of the mean. The asterisks indicate a significant difference between the two groups (*t*-test or Mann-Whitney test).^*^*p* ≤ 0.05; ^**^*p* ≤ 0.01; ^***^*p* ≤ 0.001; ^****^*p* ≤ 0.0001. pH H_2_O: pH of the soil in water; P, Phosphorus; Ca^2+^, Calcium; Mg^2+^, Magnesium; K^+^, Potassium; Na^+^, Sodium; Al^3+^, Aluminum; CTC, Cation exchange capacity; V, Base saturation; m, Saturation by aluminum; ISNa, Sodium Saturation Index; OM, Organic Matter; B, Boron; Cu, Copper; Fe, Iron; Mn, Manganese; Zn, Zinc; S, Sulfur; N, Nitrogen; and Cl-, Chlorine. ND, Not Determined.

Supplementary Table 2 List of differentially expressed peaks (mass to charge ratio—m/z) resulted from the pathway analysis using the MS Peaks to Pathway module of MetaboAnalyst 5.0, by organ—leaf or roots. Data set showing the m.z, the mode—positive or negative, the KEGG id of the matched compound, matched form, mass difference, fold change—FC, Log_2_(FC), and profile, in each one of the three scenarios evaluated: age effect—AE (control plants at 2 and 55 days under salinity stress—DAT), short-term stress—STS (control and the stress plants at 2 DAT); and long-term stress 2—LTS2 (stressed plants at 2 and 55 DAT). The differentially expressed peaks are those with an adjusted *P*-value (FDR) ≤ 0.05, of the Welch *t*-test; and Log_2_ (Fold Change) ≠ 1. LTS_Final, Effect of salt stress in the long-term discounted the age effect; Down, Downregulated; Up, Upregulated; Non-DE, Non-Differentially Expressed. If False Discovery Rate (FDR) ≤ 0.05, then Profile was called Non-DE, and FC (Fold Change) became 1.0.
