# Supplementary figures and images for: Deep Untargeted Metabolomics Analysis to Further Characterize the Adaptation Response of Gliricidia sepium (Jacq.) Walp. to Very High Salinity Stress
Source: Front Plant Sci. 2022 May 19;13:869105. doi: 10.3389/fpls.2022.869105 (PMC9161747; doi:10.3389/fpls.2022.869105)

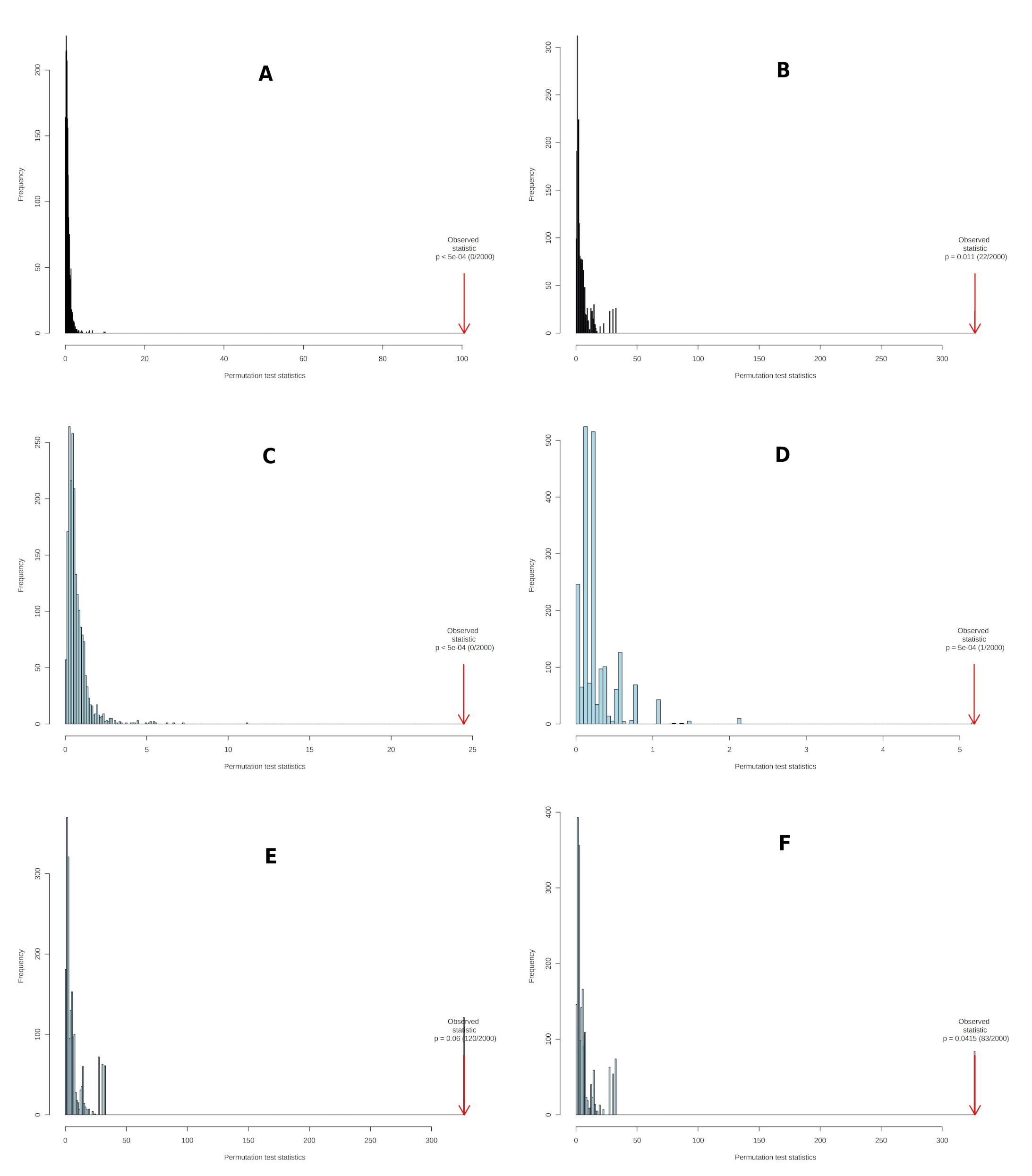

Supplement: Supplementary Figure 1 — PLS-DA permutation validation evaluated by group separation distance, applying permutation number = 2,000. From the all treatments leaves (A,C,E) and roots (B,D,F) data sets (control and stressed plants at 2 and 55 DAT). Polar-positive (A,B), polar-negative (C,D), and lipidic-positive (E,F) fractions. [file Image_1.JPEG]
